# Long‐Term Effects of Intensive Lifestyle Intervention on Cardiometabolic Outcomes in Patients With Diabetes in Real‐World Clinical Practice: A 15‐Year Longitudinal Study

**DOI:** 10.1111/1753-0407.70153

**Published:** 2025-11-05

**Authors:** Abdelrahman Khater, Marwa Al‐Badri, Tareq Salah, Shilton E. Dhaver, Karim Kibaa, Osama Hamdy

**Affiliations:** ^1^ Clinical, Behavioral and Outcome Research Department Joslin Diabetes Center Boston Massachusetts USA; ^2^ Harvard Medical School Boston Massachusetts USA

**Keywords:** cardiometabolic risk factors, diabetes, lifestyle intervention, obesity, weight management

## Abstract

**Introduction:**

We previously demonstrated that achieving and maintaining ≥ 7% weight loss at 1 year in patients with diabetes (DM), through multidisciplinary intensive lifestyle intervention (ILI) in real‐world clinical practice, predicts improvement in cardiometabolic outcomes at 5 and 10 years. In this prospective follow‐up, we report 15‐year results.

**Methods:**

We evaluated 122 patients with DM and obesity (mean age 53.2 ± 10.0 years, 68% females, 90.2% type 2 DM, mean DM duration 9.1 ± 8.6 years, mean BMI 38.4 ± 5.2 kg/m^2^) who completed a 12‐week ILI program. The cohort achieved an average weight loss of 10.7 ± 4.6 kg (−9.6%, *p* < 0.001) at 12 weeks and was divided into two groups at 1 year: group A, who maintained < 7% weight loss (47.5%) and group B, who maintained ≥ 7% weight loss (52.5%).

**Results:**

At 15 years, the cohort maintained an average weight loss of 8.6 ± 11.9 kg (−7.6%, *p* < 0.001). Group B demonstrated superior sustained weight loss (12.9 ± 12.0 kg, −11.0%) compared to group A (3.8 ± 10.0 kg, −4.0%) with *p* < 0.001 between groups. A1C in group B improved from 7.3% ± 1.1% to 6.3% ± 0.8% at 12 weeks and remained at 7.3% ± 1.5% at 15 years, while group A's A1C increased from 6.7% ± 0.9% to 7.9% ± 1.8% (*p* = 0.04 between groups). Both groups maintained LDL‐ and HDL‐cholesterol improvements, but group A experienced significant worsening of serum triglycerides.

**Conclusions:**

ILI in real‐world clinical practice that results in ≥ 7% weight loss at 1 year in patients with DM and obesity is associated with continued improvement in cardiometabolic outcomes at 15 years.

## Graphical abstract

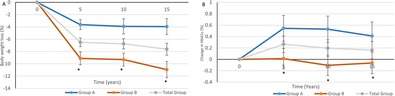

**Summary**

- Intensive lifestyle intervention (ILI) improves cardiometabolic risk factors in patients with diabetes and obesity. Maintaining ≥ 7% weight loss at 1 year following 12‐week ILI predicts sustained long‐term outcomes.
- This 15‐year study, the longest follow‐up in non‐surgical ILI for this population, demonstrates significant weight and A1C improvements in real‐world practice.
- The findings support the Look AHEAD study and highlight ILI's critical role in long‐term diabetes and obesity care, potentially influencing clinical guidelines to emphasize lifestyle interventions for improved health outcomes.

## 1 Introduction

Diabetes is a chronic disease with an alarming increase in the incidence and prevalence across the globe [1, 2]. Approximately 537 million adults today have diabetes and it is expected to affect 783 million people by 2045 [3]. From a public health perspective, healthcare expenditure on diabetes sums to over 966 billion US dollars worldwide [3]. Specifically in the US, expenditure in 2022 amounted to about 413 billion US dollars [4]. From a patient's perspective, living with diabetes increases risk for many serious complications, including cardiovascular disease, nephropathy, neuropathy, and retinopathy [5, 6, 7, 8, 9]. So far, cardiovascular disease is the most common cause of death in patients with diabetes [5, 10]. Thus, a key feature of diabetes management is to reduce cardiovascular risk factors. Obesity is a common finding in patients with diabetes and is a major risk factor for cardiovascular disease [5]. While obesity is traditionally more associated with type 2 diabetes (T2D), a higher‐than‐expected and continuously increasing prevalence of overweight or obesity is also found in patients with type 1 diabetes (T1D) [11, 12]. Lifestyle modifications involving diet, exercise, and behavior modifications are of fundamental importance in the management of patients with diabetes who are overweight or obese [13, 14].

Previous studies have demonstrated that intensive lifestyle intervention (ILI) caused a reduction in the development of diabetes for those without the disease and improved cardiovascular risk factors in those with T2D [15, 16]. While the Look AHEAD study did not find a significant reduction in cardiovascular events for those who underwent ILI [17], a follow‐up analysis stratifying participants according to the magnitude of weight loss and regain showed that greater weight loss resulted in greater improvements in A1C, systolic blood pressure, triglycerides, and HDL‐cholesterol at 1 and 4 years [18]. Moreover, those who had greater initial weight loss were shown to have greater improvements in A1C compared to those with no or small initial weight loss [18]. Meanwhile, greater weight loss in the first year was also found to be an independent predictor of long‐term weight loss in the Diabetes Prevention Program [19]. We previously demonstrated that long‐term weight loss following a 12‐week ILI in real‐world clinical practice is possible and can be predicted by the ability to achieve ≥ 7% weight loss at 1 year [20, 21]. A 5 and 10‐year follow‐up demonstrated sustained improvement in A1C and lipid profile [20, 21]. Moreover, ≥ 7% weight loss at 1 year was also associated with a decreased incidence of diabetic nephropathy at 10 years but was not associated with a similar reduction in the incidence of other microvascular or macrovascular complications [21]. A retrospective study of our cohort also demonstrated improvement in hepatic steatosis index levels and total daily insulin requirements in participants with T1D and obesity at 1 year following 12 weeks of ILI [22]. In this longitudinal study of the same total cohort of T1D and T2D, we evaluate long‐term changes in cardiometabolic outcomes at 15 years of follow‐up.

## 2 Methods

### 2.1 The Intensive Lifestyle Intervention (ILI) Program

A detailed description of our ILI program was previously published [20, 23]. To summarize, the Weight Achievement and Intensive Treatment (Why WAIT) program is a 12‐week multidisciplinary program for weight reduction and diabetes management designed and implemented at Joslin Diabetes Center in Boston, Massachusetts since September 2005. The Why WAIT program enrolled participants with type 1 or type 2 diabetes with a body mass index (BMI) between 30 and 45 kg/m^2^. Participants underwent a comprehensive evaluation by a multidisciplinary team including diabetologists, registered dieticians (RD), clinical exercise physiologists (EP), and psychologists or social workers. Eligible patients were then enrolled in groups of 10–15 participants each.

### 2.2 Study Design

Participants were assigned to one of two groups based on weight loss maintenance 1 year after participation in the Why WAIT program. Group A included participants who maintained < 7% weight loss, and Group B included participants who maintained ≥ 7% weight loss at 1 year. The rationale behind choosing the 1 year mark was due to the increased frequency of weight regain within a short period of time after the intensive period of intervention [20]. Beyond the first year, all analyses were done prospectively.

### 2.3 Study Subjects

**FIGURE 1:**
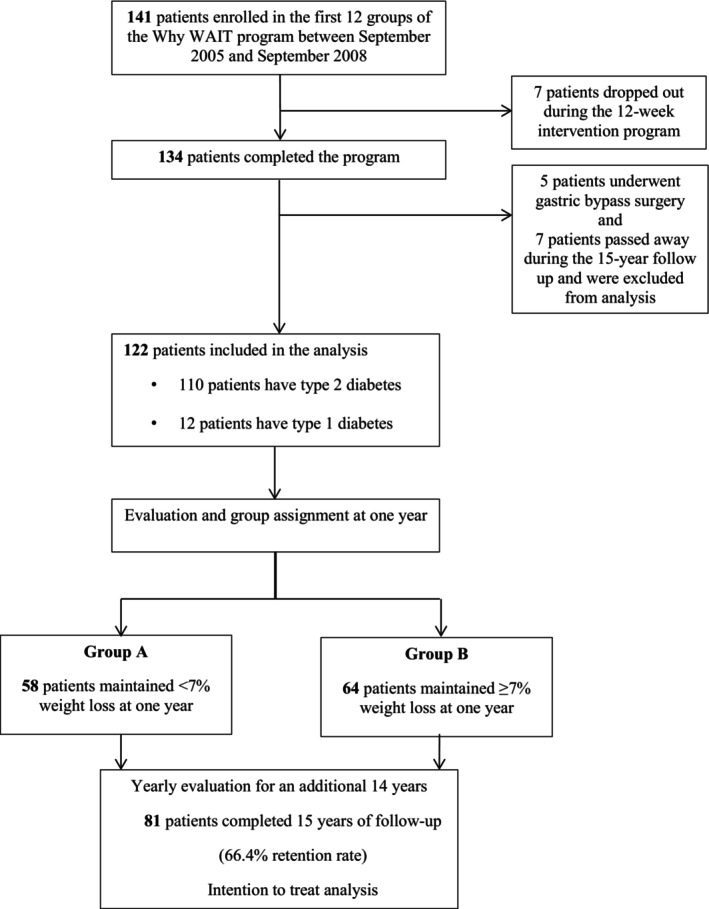
Flow of participants during the 15‐year follow up after intensive lifestyle intervention using the Why WAIT model.

We enrolled 141 participants in the Why WAIT program between September 2005 and September 2008. For the previous 5‐year outcomes analysis, 12 participants were excluded: 7 for dropping out and 5 for undergoing bariatric surgery during the follow‐up period [20]. In the previous 10‐year outcomes analysis, one additional participant passed away and was excluded. At this 15‐year outcomes analysis, 6 additional participants passed away and were also excluded. Therefore, we report data for a total of 122 participants who completed the full 15 years of follow up (Figure 1). The 15‐year follow‐up data includes 81 participants with clinic visits and direct assessments. For the remaining participants, data were extracted from electronic medical records or, when missing, carried forward using the last observation carried forward (LOCF) method. Although we did not conduct a formal sensitivity analysis limited to the 81 participants with complete 15‐year follow‐up, the use of LOCF was applied consistently and transparently to preserve sample size and analytical validity.

Follow‐up measurements were done during the participants' regular visits to the Joslin clinic over 15 years. Measurements during these visits included weight, height, blood pressure, A1C, lipid profile, blood urea nitrogen (BUN), and urinary albumin/creatinine ratio (UACR).

### 2.4 Study Endpoints

Primary endpoints for this study were change in body weight and change in A1C at 15 years. Secondary endpoints were changes in lipid profile and blood pressure at 15 years. All included data were assessed at least annually. If data were missing, the most recent data were carried forward.

### 2.5 Statistical Analysis

Tests of group differences were done using the intention‐to‐treat method. As clinic visits during the 15‐year follow‐up were not strictly scheduled every 3 months, an approximation of each visit was done to the nearest 3‐month timeline. No evidence was found that missing data was dependent on the study group. Chi‐squared test, Fisher exact test, and two‐sample *t*‐tests were used to compare baseline characteristics versus endpoints in the two study groups. All continuous variables are presented as mean ± standard deviation (SD) for consistency and ease of interpretation. While some variables (e.g., triglycerides and urinary albumin‐to‐creatinine ratio) may not follow a normal distribution, our primary outcomes (weight and HbA1c) are normally distributed. This approach aligns with our intention‐to‐treat (ITT) analysis strategy, which included all randomized participants regardless of adherence or dropout.

All statistical analyses were performed using STATA/SE 17.0 for Windows (StataCorp, College Station, Texas, USA 2021). In all tests, *p* < 0.05 was considered statistically significant.

## 3 Results

Group A included 58 patients (47.5%) and Group B included 64 patients (52.5%). At baseline, no statistically significant differences were found between the two groups in terms of age, gender, diabetes type, duration of diabetes, A1C, blood pressure (systolic and diastolic), lipid profile (total cholesterol, LDL‐cholesterol, HDL‐cholesterol and triglycerides), kidney function (serum creatinine, BUN and UACR) or smoking status. Group B had a higher BMI compared to Group A at baseline (39.4 ± 0.6 kg/m^2^ vs. 37.2 ± 0.7 kg/m^2^, *p* = 0.018). Baseline characteristics are described in Table 1.

**TABLE 1:** Baseline characteristics of the participants in the real‐world intensive lifestyle intervention.

	All participants	Group A (*n* = 58)	Group B (*n* = 64)	*p*
Age (years)	53.2 ± 10.0	52.7 ± 8.9	53.6 ± 10.9	0.636
Female sex *n* (%)	83 (68.0)	43 (74.1)	40 (62.5)	0.169
Duration of diabetes (years)	9.1 ± 8.6	9.0 ± 9.3	9.2 ± 7.9	0.912
Body weight (kg)	110.1 ± 19.4	104.2 ± 17.0	115.4 ± 20.0	0.0012^a^
Body Mass Index (kg/m^2^)	38.4 ± 5.2	37.2 ± 5.2	39.4 ± 5.1	0.018^a^
A1C (%)	7.4 ± 1.2	7.5 ± 1.4	7.3 ± 1.1	0.513
Systolic BP (mmHg)	128.0 ± 14.3	126.1 ± 13.5	129.7 ± 15.0	0.169
Diastolic BP (mmHg)	76.0 ± 8.1	76.0 ± 8.6	76.0 ± 7.6	0.991
Total Cholesterol (mg/dl)	168.2 ± 31.5	167.4 ± 29.8	169.0 ± 33.2	0.780
LDL‐Cholesterol (mg/dl)	101.0 ± 29.2	99.0 ± 27.6	102.7 ± 30.7	0.491
HDL‐Cholesterol (mg/dl)	43.7 ± 10.1	45.0 ± 10.1	42.5 ± 10.0	0.178
Triglycerides (mg/dl)	137.8 ± 74.3	129.6 ± 61.4	145.1 ± 84.0	0.245
Serum Creatinine (mg/dl)	0.91 ± 0.2	0.90 ± 0.2	0.91 ± 0.2	0.713
Blood Urea Nitrogen (mg/dl)	17.2 ± 5.5	16.8 ± 4.8	17.6 ± 6.1	0.374
Urinary Microlalbumin/Creatinine Ratio (μg/mg)	27.0 ± 53.6	22.5 ± 26.4	31.0 ± 70.0	0.409
Smoker, yes *n* (%)	32 (26.2)	13 (11.0)	19 (29.7)	0.362

*Note:* Data are mean ± SD or *n* (%). Total group *N* = 122. Group A *n* = 58 (participants maintained < 7% weight loss at 1 year). Group B *n* = 64 (participants maintained ≥ 7% weight loss at 1 year). *p* value for between groups using student's *t*‐test or Pearson's chi squared test.

Abbreviations: HDL, high‐density lipoprotein; LDL, low‐density lipoprotein.

^a^ Statistically significant.

### 3.1 Cardiometabolic Outcomes

**FIGURE 2:**
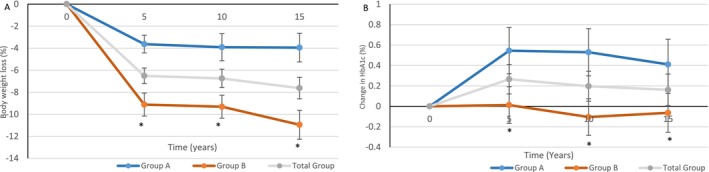
Percentage weight loss and change in A1C over 15 years in response to intensive lifestyle intervention in a real‐world clinical practice. (A) Percentage weight loss. (B) Change in A1C. *p‐value < 0.05 between groups.

At the end of the 12‐week program, the entire cohort lost 10.7 ± 4.6 kg (−9.6%, *p* < 0.001) and maintained weight loss of 7.4 ± 9.0 kg at 5 years (−6.5%, *p* < 0.0001) and 7.5 ± 10.1 kg at 10 years (−6.7%, *p* < 0.001). At 15 years, average weight loss in the entire cohort was 8.6 ± 11.9 kg (−7.6%, *p* < 0.001). Group A maintained an average weight loss of 3.9 ± 6.5 kg (−3.6%) at 5 years, 3.8 ± 9.4 kg (−3.9%) at 10 years, and 3.8 ± 10.0 kg (−4.0%) at 15 years. Group B maintained an average weight loss of 10.6 ± 9.8 kg (−9.1%) at 5 years, 10.8 ± 9.6 kg (−9.3%) at 10 years, and 12.9 ± 12.0 kg (−11.0%) at 15 years. At 15 years, weight loss in group B remained significantly higher than in group A (*p* < 0.001) (Figure 2A).

At the end of the 12‐week program, the entire cohort had an A1C reduction of −0.9% ± 1.0% (*p* < 0.001). However, A1C reduction was not maintained at 5, 10, or 15 years for the entire cohort. In group A, A1C reduced from 7.5% ± 1.4% to 6.7% ± 0.9% at 12 weeks but rebounded back to 8.04% ± 1.87% at 5 years, 8.03% ± 1.90% at 10 years, and was 7.9% ± 1.8% at 15 years. In group B, A1C decreased from 7.3% ± 1.1% to 6.3% ± 0.8% at 12 weeks, and was 7.4% ± 1.6% at 5 years, 7.2% ± 1.4% at 10 years, and 7.3% ± 1.5% at 15 years (*p* = 0.04 between groups). The trend of A1C changes compared to baseline over the 15 years is shown in Figure 2B.

**FIGURE 3:**
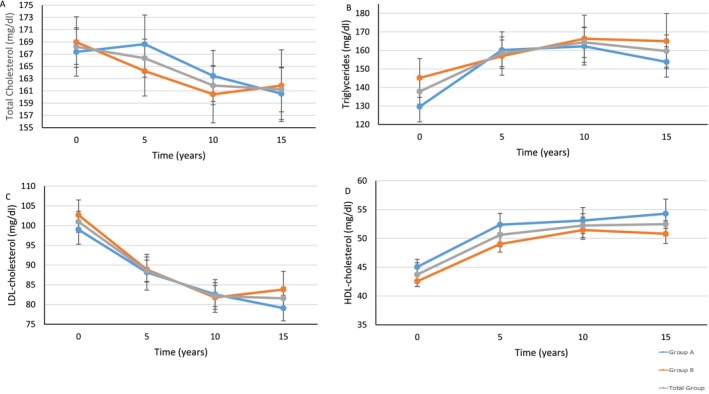
Lipid profile trend over 15 years in response to intensive lifestyle intervention in a real‐world clinical practice. (A) Total cholesterol; (B) Triglycerides; (C) LDL‐cholesterol; (D) HDL‐cholesterol.

Both groups maintained significant improvements in LDL‐ and HDL‐cholesterol at 15 years compared to baseline, but no significant change was found between groups. Group A had significant worsening of their serum triglycerides at 15 years compared to baseline (152.8 ± 62.5 mg/dL vs. 129.6 ± 61.4 mg/dL, *p* = 0.028), while no significant changes were seen in total cholesterol, serum creatinine, BUN, or UACR in either group compared to baseline. Lipid profile changes are seen in Figure 3A–D. Both groups had no significant changes in blood pressure at 15 years compared to baseline. Changes in cardiometabolic outcomes after the initial 12 weeks of the ILI program and at 5, 10, and 15 years are shown in Table 2.

**TABLE 2:** Changes from baseline in metabolic parameters after the 12‐week intensive lifestyle intervention and at 5, 10, and 15 years follow‐up in real‐world clinical practice.

	All participants				Group A				Group B				*p*
	12 weeks	5 years	10 years	15 years	12 weeks	5 years	10 years	15 years	12 weeks	5 years	10 years	15 years	
Body weight (kg)	99.4 ± 17.5***	102.7 ± 18.6***	102.6 ± 20.1***	101.5 ± 20.3***	96.4 ± 16.4	100.3 ± 16.8***	100.4 ± 19.9**	100.4 ± 20.4**	102.1 ± 18.1	104.8 ± 20.1***	104.6 ± 20.3***	102.5 ± 20.2***	0.58
HbA1c (%)	6.49 ± 0.88***	7.69 ± 1.74	7.61 ± 1.71	7.58 ± 1.70	6.71 ± 0.87***	8.04 ± 1.87*	8.03 ± 1.9*	7.91 ± 1.83	6.3 ± 0.84***	7.36 ± 1.56	7.24 ± 1.40	7.28 ± 1.53	0.04
Systolic BP (mmHg)	121.6 ± 13.3***	126.2 ± 15.5	127.5 ± 16.0	130.0 ± 16.3	121.7 ± 12.0*	126.7 ± 17.0	129.3 ± 17.8	130.7 ± 16.5	121.6 ± 14.4**	125.7 ± 14.2	125.8 ± 14.1	129.3 ± 16.3	0.64
Diastolic BP (mmHg)	73.5 ± 9.4**	74.4 ± 9.1	74.3 ± 9.2	73.9 ± 9.0*	74.5 ± 7.3	74.8 ± 9.1	74.7 ± 8.9	75.4 ± 8.3	72.5 ± 10.9*	73.9 ± 9.1	73.9 ± 9.5	72.6 ± 9.4*	0.08
Total cholesterol (mg/dl)	149.3 ± 31.0***	166.3 ± 34.3	161.9 ± 34.7	161.2 ± 40.3	152.5 ± 27.7***	168.6 ± 36.2	163.4 ± 31.7	160.6 ± 32.1	146.6 ± 33.6***	164.3 ± 32.6	160.5 ± 37.4	161.9 ± 46.8	0.86
LDL cholesterol (mg/dl)	88.3 ± 28.6***	88.5 ± 29.9***	82.1 ± 29.3***	81.6 ± 31.6***	88.8 ± 26.6***	88.1 ± 34.8*	82.9 ± 28.7***	78.8 ± 24.7***	87.9 ± 30.5***	88.9 ± 25.4***	81.8 ± 30.1***	83.8 ± 36.9***	0.4
HDL cholesterol (mg/dl)	42.3 ± 10.2	50.6 ± 15.5***	52.2 ± 17.6***	52.5 ± 18.6***	44.4 ± 10.7	52.4 ± 15.1***	53.1 ± 17.4***	54.3 ± 19.3***	40.5 ± 9.5	49.0 ± 15.8***	51.5 ± 18.0***	50.8 ± 18.0***	0.31
Triglycerides (mg/dl)	100.7 ± 49.3***	158.5 ± 78.9**	164.4 ± 90.4***	159.6 ± 96.2*	101.8 ± 48.0***	157.7 ± 73.2**	162.9 ± 77.4**	153.8 ± 62.4*	99.7 ± 50.8***	156.9 ± 82.8	166.3 ± 101.7*	165.0 ± 119.1	0.51
Serum creatinine (mg/dl)	0.91 ± 0.18	0.86 ± 0.25**	0.86 ± 0.25*	0.94 ± 0.52	0.92 ± 0.20	0.86 ± 0.25	**0.87** ± **0.28**	**0.98** ± **0.70**	**0.90** ± **0.16**	**0.86** ± **0.24** *	**0.86** ± **0.22** ^**^	0.91 ± 0.28	0.47
Blood urea nitrogen (mg/dl)	17.1 ± 5.3	17.9 ± 7.2	17.8 ± 6.0	19.0 ± 7.7**	17.6 ± 5.2	17.9 ± 6.1	18.3 ± 5.9*	19.5 ± 8.3*	16.8 ± 5.5	18.0 ± 8.2	17.4 ± 6.1	18.6 ± 7.2	0.69
Urinary microalbumin/creatinine ratio (μg/mg)	16.4 ± 23.0*	47.1 ± 186.2	79.2 ± 255.83*	95.1 ± 510.9	17.0 ± 22.9*	34.0 ± 62.6	106.5 ± 308.7*	49.8 ± 145.3	15.9 ± 23.2	59.2 ± 251.9	53.9 ± 194.1	137.9 ± 698.5	0.31

*Note:* All continuous variables are reported as mean ± SD. This consistent reporting format was chosen to align with the primary outcomes and ITT analytic strategy, though some variables may not be strictly normally distributed. Total group *N* = 128. Group A *n* = 58 (participants maintained < 7% weight loss at 1 year). Group B *n* = 64 (participants maintained > 7% weight loss at 1 year).

Abbreviation: NS, non‐significant.

* *p* < 0.05.

** *p* < 0.01.

*** *p < 0.001* compared to baseline. *p*‐value for Group A versus Group B at 15 years.

### 3.2 Anti‐Hyperglycemic Medications and Insulin

No significant differences were found between groups at 15 years in regard to use of sulfonylureas, dipeptidyl peptidase‐4 (DPP) inhibitors, glucagon‐like peptide‐1 receptor agonists (GLP‐1 RA), thiazolidinediones (TZD), short‐acting insulin, or long‐acting insulin. A statistically significant difference in metformin use was found between groups at 15 years (*p* = 0.038).

## 4 Discussion

Long‐term weight loss maintenance is often a substantial challenge, particularly for people with diabetes and obesity. To the best of our knowledge, this is the longest follow‐up study of weight management in participants of non‐surgical intensive lifestyle intervention for patients with diabetes and obesity in real‐world clinical practice. This 15‐year follow‐up analysis provides insight into factors that may predict long‐term maintenance of weight loss and associated cardiometabolic outcomes. The entire cohort lost an average of 7.6% of their initial body weight at 15 years, demonstrating that weight loss can be maintained for a long period of time. In particular, participants who lost and maintained ≥ 7% weight loss at 1 year (Group B) were able to maintain an average of 11% weight loss at 15 years. This was significantly more than participants who maintained < 7% weight loss at 1 year (Group A), as their average weight loss at 15 years was 4%. This was consistent with our findings at 5 and 10 years. Interestingly, weight loss remained stable between 10 and 15 years in Group A, while Group B lost more weight during that same period.

Moreover, a significantly lower A1C was found in group B compared to Group A at 15 years (7.3% vs. 7.9%, *p* = 0.04). Group B demonstrated a slight non‐significant reduction in A1C (−0.06%) compared to baseline, while Group A demonstrated a significant increase in A1C (+0.41%) compared to baseline, indicating the value of maintaining a higher magnitude of weight loss. Previous studies showed an increase in insulin sensitivity following a short‐term weight loss [24, 25], however, this association 15 years later is unclear. We have previously demonstrated that A1C reduction was greater in participants with higher baseline A1C when adjusted for the magnitude of weight loss [26], which can be of significant importance when discussing with patients their clinical expectations after weight loss.

Other cardiometabolic outcomes, including LDL‐ and HDL‐cholesterol, improved for both groups after 15 years, which was consistent with our earlier analyses [20, 21]. No significant changes in blood pressure were observed, which were also consistent with our findings at 5 and 10 years [20, 21]. Our results are also in‐line with the Look AHEAD trial analysis demonstrating an association between greater improvements in A1C and the magnitude of weight loss at 1 and 4 years [18]. While they demonstrated an association between larger weight loss and improvements in SBP, HDL‐cholesterol, and triglycerides, our analysis at a longer period of 15 years did not find any significant difference in these parameters between groups. While it was expected that participants who maintained weight loss for a longer duration may reduce antihyperglycemic medications, our analysis found no statistical difference in the use of oral antihyperglycemic medications or insulin, except metformin, between the two groups. This may carry little clinical significance considering the longer period of follow‐up and the progression of the disease.

Furthermore, a post hoc analysis of the Look AHEAD trial demonstrated a significantly lower risk of cardiovascular events in patients who maintained a glucose time‐in‐range (TIR) of > 50% compared to those with TIR of ≤ 50% [27]. While our study did not include an analysis of daily blood glucose values for not being collected or glucose data from Continuous Glucose Monitoring (CGM), future studies utilizing CGM technology could examine the relationship between sustained weight loss and TIR, providing a more comprehensive understanding of glycemic variability and control over time.

This study has several limitations. First, the absence of a control group undergoing standard diabetes care. However, the main aim of this study was not to evaluate the value of long‐term weight loss, but to compare cardiometabolic outcomes between participants who sustained weight loss versus those who did not after the same ILI in real‐world clinical practice. Second, many of the measured parameters in this study were surrogate biomarkers of cardiometabolic disease, and thus may or may not reflect a true improvement in cardiovascular outcomes. Future research should incorporate cardiovascular events (e.g., myocardial infarction, stroke, hospitalization) via linkage with medical records or validated self‐reports to better assess the clinical impact of long‐term weight loss maintenance. Third, the setting of this study was restricted to a single tertiary care diabetes center, thus resource availability may not be available in most primary care settings. Additionally, the ILI program requires significant time commitment and financial resources from participants, potentially limiting the generalizability of the results. An additional limitation of our study is the inability to perform gender‐stratified analyses. Although 68% of participants were female—many likely transitioning through menopause during follow‐up—we did not collect menopausal or hormonal status data in a standardized way. Subgrouping by gender in addition to weight loss classification would have resulted in small and uneven groups, limiting statistical power. Furthermore, the variability in visit timing and missing data inherent to real‐world settings posed additional challenges. Future studies should aim to incorporate gender‐specific analyses and consider the role of hormonal changes in long‐term cardiometabolic outcomes. Finally, considering the long‐term follow‐up of 15 years, newer antihyperglycemic medications were introduced and used by our patients (e.g., SGLT‐2 inhibitors, longer acting GLP‐1 RA). These medications have a positive impact on weight loss and cardiovascular outcomes in patients with an established history of atherosclerotic cardiovascular disease [28]. Although it is valuable to know the impact of those medications, it was beyond the capacity of this study to evaluate the impact of each individual or group of medications. Other dietary changes during that period, and especially during the COVID‐19 pandemic, might have an impact on lifestyle changes and their outcomes.

In conclusion, we demonstrate that significant weight reduction can be maintained for 15 years in real‐world clinical practice after a short period of ILI. Maintenance of ≥ 7% weight loss at 1 year in patients with DM and obesity is associated with continued improvement in cardiometabolic outcomes at 15 years of follow‐up.

## Author Contributions

A.K. collected data, conducted statistical analysis, and drafted the manuscript. M.A. collected data, conducted statistical analysis, reviewed and edited the manuscript. T.S. collected data, and reviewed and edited the manuscript. S.E.D. collected data, conducted statistical analysis, and reviewed and edited the manuscript. K.K. collected data, and reviewed and edited the manuscript. O.H. directs the Why WAIT program, designed the study, supervised the work, and reviewed and edited the manuscript. A.K. and O.H. are guarantors of this work and take responsibility for its integrity and the accuracy of data analysis. All authors approved the final version of the manuscript.

## Ethics Statement

The study was reviewed and approved by Joslin Diabetes Center's Committee on Human Studies (IRB approval ID# STUDY00000056) with a waiver of informed consent.

## Conflicts of Interest

O.H. receives research support from Eli Lilly and Novo Nordisk and serves on an advisory board for Abbott Nutrition. None of these entities supported this research in part or total. A.K., M.A., T.S., S.D. and K.K. have no disclosures relevant to this work.

## Data Availability

The data contained in this manuscript are held at the Joslin Diabetes Center clinical research center.
